# Clinical significance of serum soluble death receptor 5 concentration in locally advanced non-small cell lung cancer patients

**DOI:** 10.3892/ol.2014.2237

**Published:** 2014-06-11

**Authors:** XINSHUANG YU, JUAN DU, CHUNJUAN ZHAI, JIANDONG ZHANG, GUANGYUN LI, WEI DONG, DEGUO XU, FENGJUN LIU, ZHEN LIU, YUAN TIAN, MEIJUAN SONG, YING JU, BAOSHENG LI

**Affiliations:** 1 Department of Radiation Oncology, Shandong Provincial Qianfoshan Hospital, Shandong University, Shandong 250014, P.R. China; 2 Sixth Department of Radiation Oncology, Shandong Cancer Hospital, Jinan, Shandong 250117, P.R. China; 3 Central Laboratory, Shandong Provincial Qianfoshan Hospital, Shandong University, Shandong 250014, P.R. China; 4 Department of Cardiology, Shandong Provincial Hospital Affiliated to Shandong University, Shandong University, Jinan, Shandong 250014, P.R. China; 5 Department of Clinical Laboratory, Shandong Provincial Hospital Affiliated to Shandong University, Shandong University, Jinan, Shandong 250014, P.R. China

**Keywords:** soluble death receptor 5, non-small cell lung cancer, chemoradiotherapy

## Abstract

There is an urgent requirement for the identification of suitable biomarkers for the diagnosis and prognosis of non-small cell lung cancer (NSCLC). The present study aimed to measure the levels of serum soluble death receptor 5 (sDR5) in patients with locally advanced stage III NSCLC, and to evaluate its diagnostic and prognostic significance in these patients. The sDR5 concentrations were evaluated by the enzyme-linked immunosorbent assay method in 50 healthy controls and 122 patients with locally advanced stage III NSCLC [including 57 adenocarcinoma (ADC) and 65 squamous cell carcinoma (SCC) patients], before and after concurrent chemoradiotherapy. It was found that the pretreatment sDR5 levels in patients with NSCLC were higher than the sDR5 levels of healthy controls (P<0.001). However, no significant difference in the sDR5 levels was observed between the ADC and SCC subgroups (P=0.874). According to multiple clinical classifications, a significant increase in the pretreatment serum sDR5 levels could be observed in IIIB-stage patients compared with IIIA-stage patients (P=0.009). Patients with a tumor burden >3 cm had higher pretreatment sDR5 concentration than those with a tumor burden ≤3 cm (P=0.026). Additionally, T4-stage patients had significantly higher pretreatment sDR5 levels compared with those of T1-stage patients (P<0.001). There were no significant differences between pre- and post-treatment sDR5 concentrations in the total NSCLC patient group (P=0.462), ADC subgroup (P=0.066) and SCC subgroup (P=0.052). Furthermore, when patients were divided according to therapeutic response, the pretreatment sDR5 levels in the responder patients were significantly lower compared with those of the non-responders (P<0.001). Further survival analysis showed that the patients whose pretreatment sDR5 levels were ≤14 pg/ml (cutoff value, 14 pg/ml) had a longer progression-free survival (PFS) time than patients with sDR5 levels >14 pg/ml. However, no correlation was observed between the post-treatment sDR5 levels and therapeutic response or PFS time. To the best of our knowledge, the present study results provide the first evidence that the pretreatment serum levels of sDR5 may be a useful biomarker for the diagnosis, prediction and prognosis of patients with locally advanced stage III NSCLC.

## Introduction

Lung cancer is one of the leading causes of cancer-related mortality worldwide, due to its late diagnosis (1), and non-small cell lung cancer (NSCLC) accounts for >85% of all lung cancer cases (2). The majority of patients present with the advanced stage, by which time treatment is not able to cure the disease (3). Concurrent chemotherapy plus thoracic radiotherapy have become the standard therapeutic regimens for locally advanced NSCLC (4,5). However, numerous patients demonstrate a poor, or occasionally, no response to these therapies, with prompt progression of the disease. Serum biomarkers are increasingly being evaluated for their ability to facilitate early diagnosis and predict therapeutic response, which may aid in the development of patient-tailored treatment strategies for NSCLC.

Apoptosis serves as a natural barrier to cancer development. Accumulated data (6) demonstrate that alterations in the expression of death ligands and their receptors are associated with carcinogenesis. FAS/FASL (CD95/CD95 ligand) and tumor necrosis factor (TNF)-related apoptosis-inducing ligand (TRAIL)/TRAIL receptor (TRAIL-R) are two of the important death receptor-ligand systems that have been demonstrated to be involved in processes of various human tumors (7–10). The TRAIL/TRAIL-R system has been shown to selectively induce apoptosis in various tumor cells but not in normal cells. Due to this unique merit, there is a growing interest in studying the significance of the TRAIL/TRAIL-R system in various types of cancer (11).

TRAIL has five receptors which have been identified, including two death receptors (DR4 and DR5), two decoy receptors (DcR1and DcR2) and soluble receptor osteoprotegerin (12). The binding of TRAIL to its transmembrane receptors DR4 and DR5 can activate the downstream caspase cascade and finally induce the development of apoptosis (13). DR5 has been demonstrated to possess the highest affinity with TRAIL and play the most important role in TRAIL-inducing apoptosis (14). Our previous data (15) showed that sDR5 levels played a vital role in hepatitis B virus (HBV)-induced liver damage, and serum sDR5 levels may be a useful prognostic indicator of HBV infection. However, the significance of serum sDR5 levels in NSCLC patients has not yet been elucidated. In the present study, we investigated serum sDR5 concentrations in patients with locally advanced stage III NSCLC, and analyzed the correlation with clinical parameters, such as histopathological type, stage of disease, tumor burden and progression-free survival (PFS). Therefore, the present study aimed to evaluate its predictive and prognostic significance in patients with locally advanced stage III NSCLC.

## Materials and methods

### Patients

In total, 122 patients with locally irresectable stage III NSCLC, including 57 adenocarcinoma (ADC) patients and 65 squamous cell carcinoma (SCC) patients, who visited Shandong Provincial Qianfoshan Hospital (Jinan, China) between January 2010 and July 2011, were selected as candidates. All patients were histologically or cytologically confirmed. Any patient who received surgery for lung cancer was not eligible to participate. Case samples were collected at two time points: Before treatment and after concurrent chemoradiotherapy (CRT). The control group consisted of 50 healthy volunteers. All of the healthy controls were age and gender-matched with the patients. All patients were staged according to the seventh edition of the American Joint Committee on Cancer (AJCC) system for lung cancer (16). TNM (tumor nodes metastasis) staging method was used (17). Tumor response was measured using the Response Evaluation Criteria In Solid Tumors (RECIST) criteria (18).

Patients were treated with 60-Gy radiotherapy administered as 2 Gy/day for 5 days a week over ~6 weeks with platinum-doublet chemotherapy. The therapeutic dose was adjusted according to individual conditions. Follow-up was performed from the start of CRT to last confirmation of regression, including physical examination, blood chemistry, ultrasound of the abdomen and lymph node X-ray of the chest or CT scanning, scintigraphy of the skeleton and brain CT scanning if necessary.

This study was approved by the ethics committee of Shandong Provincial Qianfoshan Hospital. Written informed consent was provided by all patients and controls before sample collection. All serum samples were stored at −80°C until batch analysis by enzyme-linked immunosorbent assay (ELISA).

### Method

The concentration of sDR5 was detected by using a solid phase sandwich ELISA kit (cat. no. IB-17792; Human DR5 ELISA kit; Shanghai Jianglai Biotech, Shanghai, China) according to the manufacturer’s instructions, with the detection range from 2 to 70 pg/ml. The value of absorbance at 450 nm was utilized to draw the standard curve and the levels of sDR5 were obtained from the curve. Each serum sample was tested in duplicate.

### Statistical analysis

Serum sDR5 concentration was expressed as the mean ± standard deviation. Differences between the two groups were analyzed by Student’s t-test. Differences between multiple groups were determined by analysis of variance or the Kruskal-Wallis test. Survival analysis and curves were established according to the Kaplan-Meier method and were compared using the log-rank test. PFS was calculated as the time between the start date of the primary treatment and the date of disease progression or the last follow-up appointment. The cutoff point was chosen according to the receiver operating characteristic (ROC) analysis. Differences were considered to be statistically significant with P<0.05. All data were analyzed using SPSS 13.0 software (SPSS, Inc., Chicago, IL, USA).

## Results

### Patient characteristics

The basic characteristics of the patients are shown in Table I. The median age of healthy controls was 48 years (range, 35–70 years) and that of NSCLC patients was 51 years (range, 36–68 years). No statistical difference was observed in gender or age between the controls and patients. The objective response rate, referring to complete responses (CRs) and partial responses (PRs), was 74%. Similarly, the objective response rate in the ADC subgroup was 61.4% (35 out of 57 patients) and in SCC subgroup was 60.0% (39 out of 65 patients), respectively.

### Detection of serum soluble DR5 levels in NSCLC patients and healthy controls

**Figure 1:**
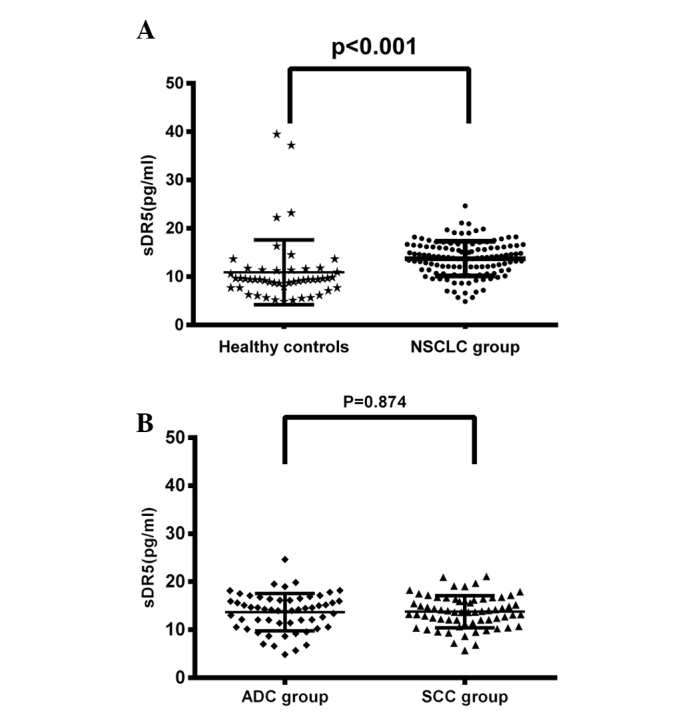
Detection of pretreatment sDR5 levels by enzyme-linked immunosorbent assay in 50 healthy controls and 122 NSCLC patients (including 57 ADC and 65 SCC patients). The groups were analyzed by Student’s t-test. (A) Pretreatment serum sDR5 levels in NSCLC patients differed from the sDR5 levels of healthy controls (P<0.001). (B) No difference in pretreatment serum sDR5 levels was identified between the ADC and SCC subgroups (P=0.874). sDR5, soluble death receptor 5; NSCLC, non-small cell lung cancer; ADC, adenocarcinoma; SCC, squamous cell carcinoma.

The pretreatment serum sDR5 levels in the healthy control group and the NSCLC group were 10.89±6.72 and 13.72±3.61 pg/ml, respectively. As presented in Fig. 1A, the pretreatment serum sDR5 levels in all patients were significantly increased compared with the sDR5 levels of healthy controls (P<0.001). However, the sDR5 levels showed no significant difference between the ADC and SCC patient groups (13.67±3.89 vs. 13.77±3.32 pg/ml; P=0.874; Fig. 1B).

### Expression of sDR5 in association with the clinical characteristics of NSCLC patients

**Figure 2:**
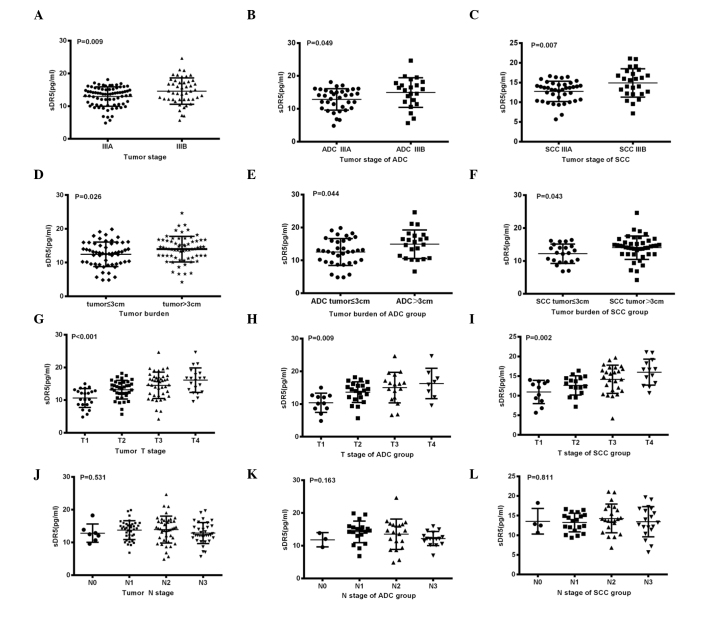
Correlation between pretreatment serum sDR5 levels and clinical characteristics of NSCLC patients. Pretreatment sDR5 levels were compared between IIIA and IIIB stage in (A) all NSCLC patients (P=0.009), (B) the ADC subgroup (P=0.049) and (C) the SCC subgroup (P=0.007). Similarly, serum sDR5 levels were compared between patients with a tumor load of ≤3 and >3 cm in (D) all NSCLC patients (P=0.026), (E) the ADC subgroup (P=0.044) and (F) the SCC subgroup (P=0.043). Serum sDR5 levels were compared among the various T stages in (G) all NSCLC patients (P<0.001), (H) the ADC subgroup (P=0.009) and (I) the SCC subgroup (P=0.002). Serum sDR5 levels were compared among N stages in (J) all NSCLC patients (P=0.531), (K) the ADC subgroup (P=0.163) and (L) the and SCC subgroup (P=0.811). The unpaired t-test was used for analyzing the difference between two groups. Differences between multiple groups were determined by analysis of variance or the Kruskal-Wallis test. sDR5, soluble death receptor 5; NSCLC, non-small cell lung cancer; ADC, adenocarcinoma; SCC, squamous cell carcinoma.

When the clinical classifications of the NSCLC patients were considered, a significant increase in pretreatment serum sDR5 levels could be observed in IIIB stage patients compared with IIIA stage patients (P=0.009; Fig. 2A). Similar results were observed between the IIIA and IIIB stage patients when patients were separated into ADC (P=0.049; Fig. 2B) and SCC (P=0.007; Fig. 2C) subgroups. Regarding the tumor burden, analysis revealed a marked increase in pretreatment sDR5 concentration in patients with a tumor load of ≤3 cm compared with patients with a load of >3 cm (12.43±0.48 vs. 13.95±0.47 pg/ml; P=0.026; Fig. 2D). Similar results were identified between the patients with different tumor burdens in the ADC subgroup (P=0.044; Fig. 2E) and SCC (P=0.043; Fig. 2F) subgroups. Pretreatment serum sDR5 levels in patients with T4 stage tumors were significantly higher than those in patients with T1 stage tumors (P<0.001; Fig. 2G). Similar results were observed between patients with T1 and T4 stage tumors in the ADC (P=0.009; Fig. 2H) an SCC (P=0.002; Fig. 2I) subgroups. However, no such correlation was found with N stage (Fig. 2J–L).

### Comparison of sDR5 levels before and after CRT

Analysis of the sDR5 concentrations before and after CRT demonstrated that there were no significant differences between pre- and post-treatment sDR5 concentrations among all NSCLC patients (P=0.462), the ADC subgroup (P=0.066) or the SCC subgroup (P=0.052), as shown in Table II.

### Change in serum DR5 levels according to clinical response after CRT

The treatment response is one of vital indices of the effectiveness of CRT in NSCLC patients. We defined patients with CRs or PRs as responders, while those with stable or progressive disease were considered non-responders, according to RECIST criteria (18). When the patients were grouped according to response to CRT, pretreatment sDR5 levels in the responder group were significantly lower than those in the non-responder group (P<0.0001; Table III). Further analysis in the ADC and SCC subgroups demonstrated the same trend (Table III). However, there was no correlation between the post-treatment sDR5 levels and clinical response (Table IV).

### Correlation between sDR5 levels and PFS time

**Figure 3:**
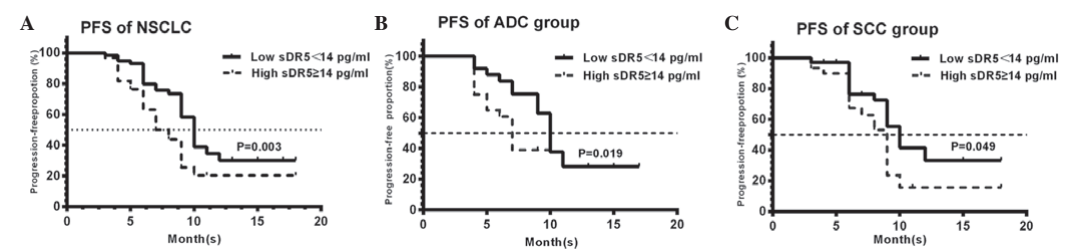
Correlation between pretreatment sDR5 levels and PFS time. The median PFS time was 8.9 months. The cutoff point of 14 pg/ml was chosen according to receiver operating characteristic analysis. Pretreatment sDR5 levels in patients with sDR5 levels of <14 and ≥14 pg/ml were compared in (A) all NSCLC patients (P=0.003), (B) the ADC subgroup (P=0.019) and (C) the SCC subgroup (P=0.049), using the Kaplan-Meier method. sDR5, soluble death receptor 5; PFS, progression-free survival; NSCLC, non-small cell lung cancer; ADC, adenocarcinoma; SCC, squamous cell carcinoma.

**Figure 4:**
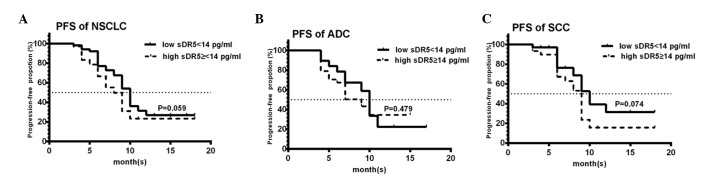
Correlation between post-treatment sDR5 levels and PFS time. The median PFS time was 8.9 months. The cutoff point of 14 pg/ml was chosen according to receiver operating characteristic analysis. Post-treatment sDR5 levels in patients with sDR5 levels of <14 and ≥14 pg/ml were compared in (A) all NSCLC patients (P=0.059), (B) the ADC subgroup (P=0.479) and (C) the SCC subgroup (0.074), using the Kaplan-Meier method. sDR5, soluble death receptor 5; PFS, progression-free survival; NSCLC, non-small cell lung cancer; ADC, adenocarcinoma; SCC, squamous cell carcinoma.

To evaluate the correlation between sDR5 levels and the outcome of patients following CRT, we calculated the PFS time of patients. At the median follow-up of 18 months (range, 3–24 months), the median PFS time was 8.9 months. Patients were then subdivided into two groups according to the sDR5 cutoff value (14 pg/ml), which was calculated by ROC analysis. In the NSCLC group, the median PFS time in patients with pretreatment sDR5 levels of >14 pg/ml was 8 months, while that of patients whose pretreatment sDR5 levels were ≤14 pg/ml was 10 months. There was a statistically significant difference in PFS time between the two groups (P=0.003; Fig. 3A). Further analysis of the ADC and SCC subgroups demonstrated the same trend (P=0.019; Fig. 3B; and P=0.049; Fig. 3C, respectively). That is, high serum sDR5 levels were associated with a lower PFS compared with low sDR5 levels, both in the ADC and SCC subgroups. However, there was no correlation between the post-treatment sDR5 levels and PFS (Fig. 4).

## Discussion

Apoptosis plays a significant role in maintaining body homeostasis. TRAIL/TRAIL-R induced apoptosis is an important regulatory pathway, which serves its potential role as a mediator of tumor immune surveillance (19). DR5 is a prominent death domain-containing receptor for TRAIL (20). Our previous study (21) showed that downregulation of DR5 was involved in the apoptosis of the HBV-related hepatoma cell line. To the best of our knowledge, the present study is the first to demonstrate that the serum levels of sDR5 may be a useful biomarker for the diagnosis and prognosis of patients with locally advanced stage III NSCLC.

In several studies, the clinical significance of DR5 expression in human tumors has been determined. Ganten *et al* (22) showed that DR5 expression was negatively associated with poor clinical outcome in breast cancer patients. Leithner *et al* (23) demonstrated that nuclear and cytoplasmic DR5 were prognostic factors in patients with NSCLC treated with chemotherapy. Zhuang *et al* (24) found that decreased DR5 expression was associated with the progression of melanoma. However, all of the above results were obtained by the immunhistochemical analysis of tumor tissues, which is an invasive immunodiagnostic method. In the present study, we used the non-invasive method, ELISA assay, to detect serum soluble DR5 levels and evaluate their diagnostic and prognostic significance in locally advanced NSCLC patients.

The current study found that pretreatment sDR5 serum levels in locally advanced stage III NSCLC patients were higher than the serum sDR5 levels of healthy controls (P<0.001). According to multiple clinical classification analysis, a significant increase in pretreatment sDR5 serum levels could be observed between IIIB and IIIA stage patients (P=0.009), and patients with T4 stage tumors had significantly higher pretreatment sDR5 levels compared with those with T1 stage tumors (P<0.001). Furthermore, patients with a tumor burden of >3 cm had higher pretreatment sDR5 concentrations compared with those with tumor burdens of ≤3 cm. The results showed that pretreatment sDR5 serum concentrations may be a usefully adjunctive factor in the diagnosis of locally advanced stage III NSCLC patients.

Further analysis found that when patients were divided according to therapeutic response (responders versus non-responders), the pretreatment sDR5 levels were significantly lower in responders compared with non-responders (P=0.007). Therefore, CRT was more effective in patients with lower pretreatment sDR5 levels than in those with higher pretreatment sDR5 levels. The results indicated that pretreatment serum sDR5 levels may aid in the development of more powerful strategies to improve the treatment efficacy for locally advanced stage III NSCLC patients.

To investigate the correlation between the sDR5 levels and the outcome of the NSCLC patients, PFS survival analysis was performed. It was found that high sDR5 serum levels were associated with a shorter PFS time compared with low sDR5 levels in NSCLC patients; patients whose pretreatment sDR5 levels were ≤14 pg/ml (cutoff value, 14 pg/ml) had an improved disease outcome compared with patients whose pretreatment sDR5 levels were >14 pg/ml. These results indicated that serum sDR5 levels may be a useful prognostic biomarker for patients with locally advanced stage III NSCLC.

At present, the cellular origin of the increased serum sDR5 levels observed in the present study is unknown. Although Yildiz *et al* detected the expression of serum sDR5 levels in metastatic colorectal cancer, the authors did not investigate the generation of serum sDR5 (9). We propose that another important death receptor, serum sFas, may originate from the tumor tissues themselves, as a correlation between sFas/CD95 serum concentration and the patient’s stage of disease has been observed (25). In the present study, it was also found that serum sDR5 levels correlated with the patient’s stage of disease and disease progression. Therefore, according to the above evidence, we hypothesize that sDR5 may be generated by the lung cancer tissue itself.

However, no correlation was identified between the post-sDR5 level and the treatment response or the PFS time in the present study. These results may be due to the fact that post-treatment sDR5 levels were affected by six weeks of CRT. Post-treatment sDR5 levels had no prognostic significance in locally advanced stage III NSCLC patients

In conclusion, pretreatment sDR5 serum concentration s may be a usefully adjunctive diagnostic index for locally advanced stage III NSCLC patients. Notably, pretreatment sDR5 levels in the patient’s serum may be a predictive and prognostic biomarker for the effectiveness of CRT in locally advanced stage III NSCLC patients.

## Figures and Tables

**Table I:** Clinical characteristics of patients.

Characteristics	n (%)	sDR5 (pg/ml)
Controls	50	10.89±6.72
Age, years	48 (35–70)^a^	
Gender		
Male	25 (50.0)	10.83±6.98
Female	25 (50.0)	10.96±6.58
Patients	122	13.72±3.61
Age, years	51 (36–68)^a^	
Gender		
Male	63 (51.4)	13.70±4.62
Female	59 (48.6)	13.73±4.46
Histopathological type		
Adenocarcinoma	57 (40.1)	13.67±3.89
Squamousl carcinoma	65 (45.8)	13.77±3.32
Stage		
IIIA	75 (61.5)	12.94±2.95
IIIB	47 (38.5)	14.62±4.03
T level		
T1	22 (15.5)	-
T2	37 (26.1)	-
T3	42 (29.6)	-
T4	21 (14.8)	-
N level		
N0	7 (5.70)	-
N1	37 (30.3)	-
N2	43 (35.2)	-
N3	35 (28.8)	-
Tumor burden, cm		
≤3	57 (47.8)	12.43±0.48
>3	65 (52.2)	13.95±0.47
Evaluation		
Total response (rate)	74 (60.6)	-
Adenocarcinoma		
CR + PR	35 (61.4)	12.67±3.58
PD + SD	22 (38.6)	15.24±3.93
Squamous carcinoma		
CR + PR	39 (60.0)	12.95±3.12
SD + PD	26 (40.0)	15.00±3.28

a Median (range).

Serum sDR5 levels were compared by Student’s t-test or analysis of variance. Results are shown as the means ± SD. Stage IIIA represents T1-2N2M0, T3N1-2M0 and T4N0-1; and stage IIIB represents T4N2M0 and T3-4N3M0, T stage and N stage are defined according to the seventh edition of the tumor-node-metastasis classification for malignant tumors (16). CR, complete response; PR, partial response; PD, progressive disease; SD, stable disease.

**Table II:** Comparasion of sDR5 level (pg/ml) before and after chemoradiotherapy.

	NSCLC	ADC group	SCC group
Pre-CRT	13.72±3.61	13.73±3.88	13.82±3.33
Post-CRT	13.39±3.39	13.32±3.73	13.46±3.08
P-value	0.462	0.066	0.052

The paired t-test was used for analyzing the differences between patients before and after treatment. There was no statistical difference in sDR5 level before and after treatment in all NSCLC patients (P=0.462), the ADC group (P=0.066) and the SCC group (P=0.052). sDR5, soluble death receptor 5; NSCLC, non-small cell lung cancer; ADC, adenocarcinoma; SCC, squamous cell carcinoma; CRT, chemoradiotherapy.

**Table III:** Pretreatment sDR5 level (pg/ml) according to treatment response.

	NSCLC	ADC group	SCC group
Responders	12.57±0.37	12.67±3.58	12.95±3.12
Non-responders	15.16±0.49	15.24±3.93	15.00±3.28
P-value	<0.0001	0.014	0.011

Student’s t-test was used for analyzing the differences in pretreatment sDR5 level according to treatment response. There was a significant difference in the sDR5 levels between the responders and non-responders in NSCLC patients (P<0.0001). The same trend was observed in the ADC and SCC subgroups (P=0.014 and P=0.011, respectively). Responders include patients with a complete or partial response, and non-responder refers to patients with stable or progressive disease. sDR5, soluble death receptor 5; NSCLC, non-small cell lung cancer; ADC, adenocarcinoma; SCC, squamous cell carcinoma.

**Table IV:** Post-treatment sDR5 levels (pg/ml) according to treatment response.

	NSCLC	ADC group	SCC group
Responders	12.97±0.32	12.93±0.48	13.01±0.45
Non-responders	14.02±0.43	13.88±0.75	14.13±0.51
P-value	0.054	0.269	0.108

Student’s t-test was used for analyzing the differences in sDR5 levels following CRT according to treatment response. No significant difference in post-treatment sDR5 levels was identified between the responders and non-responders, in any group. Responders include patients with a complete or partial response, and non-responder refers to patients with stable or progressive disease. sDR5, soluble death receptor 5; NSCLC, non-small cell lung cancer; ADC, adenocarcinoma; SCC, squamous cell carcinoma.
